# RESILIENCY AS A PROCESS: A QUALITATIVE INVESTIGATION INTO U.S. VETERANS’ EXPERIENCES AGING WITH HIV

**DOI:** 10.1093/geroni/igac059.2865

**Published:** 2022-12-20

**Authors:** Kiera Chan, Alexis Bender, Vincent Marconi, Amy Justice, Theodore Johnson, Keith McInnes, Molly Perkins

**Affiliations:** Emory University, Atlanta, Georgia, United States; Emory University, Atlanta, Georgia, United States; Emory University, Decatur, Georgia, United States; Yale University, New Haven, Connecticut, United States; Emory University, Atlanta, Georgia, United States; Boston University, Boston, Massachusetts, United States; Emory University School of Medicine, Atlanta, Georgia, United States

## Abstract

U.S. military Veterans aging with HIV represent a unique special population. The aim of this qualitative study was to investigate risk and protective factors associated with Veterans’ ability to age well with HIV. Participants included 25 Veterans (≥ age 50) participating in the Veterans Aging Cohort Study who were recruited from the Atlanta Veterans Affairs (VA) Medical Center. This study conducted semi-structured interviews, social network mapping, and sociodemographic and health surveys. Participants ranged in age from 50 to 72, with a mean age of 59. Most (80%) were male, and more than half (60%) were African American. Findings showed that many participants experienced adversity in childhood or early adulthood, including military sexual violence, childhood abuse, and non-military domestic violence, as well as harassment based on sexual identity. Many also had histories of substance abuse. Although military life provided stability for some, most experienced some form of instability after leaving the service, including financial difficulties and loss of valued military ties. Timing of diagnosis, whether in the military or after the military, impacted resiliency. Receiving an HIV diagnosis was an important turning point in participants’ lives characterized by either maladaptive (e.g., suicide ideation) or therapeutic (e.g., health promoting behaviors) coping strategies. Positive social support, including close relationships many developed with providers at the VA HIV clinic, was an important protective factor. The sample experienced cumulative life events that shaped their ability to age with HIV. Findings have important implications for interventions to promote Veterans’ ability to age well with HIV.

